# Specialty chemicals from microbes: An annotated selection of World Wide Web sites relevant to the topics in microbial biotechnology

**DOI:** 10.1111/1751-7915.12287

**Published:** 2015-04-17

**Authors:** Lawrence P Wackett

**Affiliations:** Department of Biochemistry, Molecular Biology & Biophysics, BioTechnology Institute, University of MinnesotaSt. Paul, MN, 55108, USA

## Biosynthesizing 2-hydroxybutyric acid

### http://corporate.evonik.com/en/media/focus/Pages/bacteria-syngas.aspx

Synthesis gas, that consists largely of carbon monoxide, carbon dioxide and hydrogen, has been used in a process to make the commodity chemical, 2-hydroxybutyric acid.

## Chemical production with biology

### http://cen.acs.org/articles/93/web/2015/03/Accelerating-Chemical-Production-Biology.html

This news report discusses a proposal to alter regulations of materials and products involved in using synthetic biology to make chemicals.

## Harnessing evolution for chemical production

### http://www.pnas.org/content/111/50/17803.abstract?sid=193e4e06-7d02-4b92-814c-30973c46d0d6

Genetic engineering can be complex and time consuming. This research paper describes an approach to set up microbes in a way that they increase fitness by evolving to produce higher levels of desired chemicals, in this cas naringenin and glucaric acid.

## Microbial biomanufacturing of opiods

### http://www.nature.com/nchembio/journal/v10/n10/full/nchembio.1613.html

This paper describes metabolic engineering for the microbial production benzylisoquinoline alkaloids; codeine, morphine, and hydrocordone. The microbial production of these chemicals can both decrease cost and increase security in the supply chain.

## Review on bio-based fine chemicals

### http://www.microbialcellfactories.com/content/pdf/s12934-014-0173-5.pdf

This article provides a recent review of methods and projects involving the microbial biosynthesis of industrial chemicals.

## 1,4-Butanediol from microbes

### http://www.microbemagazine.org/index.php?option=com_content&view=article&id=303:microbes-engineered-to-make-high-value-commodity-chemicals&catid=130&Itemid=249

This page put out by the American Society for Microbiology describes the bacterial production of the industrial fine chemical 1,4-butanediol.

## Blog on regulations and business issues of microbial chemical production

### https://dglassassociates.wordpress.com/about/

The commercial site is a good resource to look up regulations, licensing and technologies releated to microbially produced fuels and chemicals.

## WarpDrive Bio

### http://www.warpdrivebio.com

WarpDrive is a company devoted to actinomycete genomics for the purpose of discovering novel microbial compounds, largely for medicinal purposes.

## Metabolic engineering: Novozymes

### http://www.novozymes.com/en/about-us/our-business/industrial-biotechnology/basic-technologies/Pages/metabolic-engineering.aspx

Novozymes is the largest enzyme producing company and they also conduct metabolic engineering for producing industrial chemicals. One example is the production of hyaluronic acid.

## Metabolic engineering: Practical examples

### http://www.biotechresources.com/services-metabolic.shtml

This company website has details of practical examples of microbial engineering for the production of terpenoid compounds and amino sugars.

## Production of chemicals in *Saccharomyces*

### http://www.ncbi.nlm.nih.gov/pubmed/25238571

This recent review is focused on synthetic biology with a particular focus on chemical production using *Saccharomyces cerevisiae*.

## Production of isoprene from syngas: Patent

### http://www.google.com/patents/US20140234926

This patent describes the use of acetogenic bacteria to transform synthesis gas into the valuable product isoprene.

## Enhanced production of isoprene

### http://www.faqs.org/patents/app/20140128645

Isoprene is a valuable commodity chemical, of particular value for producing rubber for tires. Methods that enhance isoprene yields from various precursors are highly useful.

## 1,3-Propanediol market is dominated by the biological process

### http://www.marketsandmarkets.com/Market-Reports/1-3-propanediol-pdo-market-760.html

This market report discusses that 1,3-propanediol is a large-scale commodity chemical and that the main production is by a biological process developed by DuPont-Tate and Lyle.

## Rubber from microbes

### http://www.technologyreview.com/news/418159/rubber-from-microbes/

This news article describes a process developed at Genencor (now DuPont) to make the commodity chemical isoprene using engineered *E. coli* cells.

